# Valorization of Pink Salmon (*Oncorhynchus gorbuscha*) Processing By-Products: Nutritional Composition, Starch-Modified Gel Quality and Digestibility

**DOI:** 10.3390/foods15183259

**Published:** 2026-09-15

**Authors:** Meng Li, Jia Wan, Wenxuan Guan, Bo Liu, Xinda Xu, Qiancheng Zhao, Xinru Fan

**Affiliations:** 1 College of Food Science and Engineering, Dalian Ocean University, Dalian 116023, China; 2 Liaoning Provincial Marine Healthy Food Engineering Research Centre, Dalian 116023, China; 3 Dalian Key Laboratory of Marine Bioactive Substance Development and High Value Utilization, Dalian 116023, China; 4 Dalian Ruichi Food Co., Ltd., Dalian 116037, China

**Keywords:** pink salmon by-products, nutritional profile, starch-modified gel, starch, gel properties

## Abstract

Pink salmon (*Oncorhynchus gorbuscha*) processing by-products represent an underutilized resource with considerable potential for value-added applications. To evaluate the nutritional profile and investigate the effects of different starches (native and modified) on the gel properties of the pink salmon by-products, the proximate composition, amino acid profile, and fatty acid profiles of the by-products were analysed, followed by assessments of the gel texture, water-holding capacity, cooking loss, color, microstructure, and in vitro digestibility. The results demonstrated that the by-product meat possessed a high protein content and was rich in polyunsaturated fatty acids (PUFAs), with an essential amino acid-to-total amino acid (EAA/TAA) ratio of 45.82% and an n-3/n-6 ratio of 1.34, indicating favourable nutritional quality for human consumption. Although the by-product meat alone formed a relatively soft gel, the incorporation of starch markedly improved its gel properties. Among the evaluated starches, tapioca hydroxypropyl distarch phosphate (HDP) produced the greatest improvements in texture profiles and water-holding capacity while reducing cooking loss and promoting the formation of a denser and more uniform gel microstructure. Furthermore, HDP significantly enhanced the dry matter digestibility of the starch-modified gels prepared from pink salmon by-product meat. These findings demonstrate that pink salmon by-product meat can serve as a promising material for producing nutritional functional food and that HDP is an effective additive for improving both gel quality and digestibility, thereby supporting the sustainable valorization of fishery processing byproducts.

## 1. Introduction

The continued growth of the global population has led to an increasing demand for fish and fishery products worldwide. Pink salmon (*Oncorhynchus gorbuscha*) is the dominant species in commercial offshore salmon fisheries in the North Pacific Ocean [1]. Owing to its high nutritional value, particularly its abundance of high-quality protein and polyunsaturated fatty acids, it is recognized as an important commercial fish species worldwide [2,3]. Most harvested fish are processed into fillets; however, conventional filleting generates substantial quantities of by-products, accounting for approximately one-quarter of the total raw material. These by-products include heads, tails, bones, skin, frames and red meat, with the residual flesh comprising up to 50% of the total by-product stream [4]. Despite their considerable nutritional value, these materials remain underutilized and are typically directed toward low-value applications [5]. This represents a significant inefficiency in resource utilisation and a missed opportunity for the development of sustainable value-added products.

With the growing demand for seafood production, increasing attention has been directed towards the valorization of fishery processing byproducts [6,7]. Converting these materials into surimi-based products, such as fish cakes, offers an effective strategy for extending shelf life, reducing waste, and increasing the economic value of fishery products [8]. Raw materials rich in myofibrillar proteins are generally preferred for surimi manufacture because these proteins readily form cross-links that generate stable three-dimensional gel networks. However, pink salmon by-product meat contains considerable quantities of non-myofibrillar protein components, including water-soluble proteins, pigments, and lipids, all of which impair gel formation. Although several studies have demonstrated the proteins isolated from salmon by-products exhibit excellent gel-forming ability and considerable potential for the production of surimi and other gel-based foods, their industrial application remains limited because protein extraction is labour-intensive, time-consuming, and economically costly [9,10]. Consequently, there is a need to identify simple, environmentally friendly, and sustainable approaches for improving the gel-forming properties of by-product meat.

The functional properties of reconstituted surimi can be enhanced through the incorporation of various food additives. Among these, starch is widely used because of its low-cost and multifunctional properties, playing a key role in modifying gel texture [11]. During heating, starch granules absorb water and swell, exerting pressure on the surrounding protein network and thereby enhancing gel properties [12]. However, the efficacy of these improvements depends on both the botanical origin of the starch and the type of chemical modification applied [13,14]. In addition, interactions between starch and protein can influence the nutritional quality of surimi products as starch incorporation alters gel microstructure and enzyme accessibility, thereby affecting in vitro digestibility [15,16]. Although the effects of starch on the textural properties and digestibility of fish- and meat-based proteins have been extensively reported [17,18,19], comprehensive studies investigating the influence of different modified starches on pink salmon processing byproducts remain limited.

Therefore, the objective of this study was to characterize the nutritional composition, protein quality, and lipid profile of pink salmon by-product meat and to assess its suitability as a raw material for the production of pink salmon by-product meat gel. Furthermore, the effects of eight different native and modified starches on gel texture, water-holding capacity, cooking loss, color, microstructure, and in vitro digestibility were systematically investigated. The findings provide a scientific base for the efficient utilisation of by-products from pink salmon processing and support the development of sustainable, nutritious, and value-added surimi-based foods.

## 2. Materials and Methods

### 2.1. Materials

Pink salmon (*Oncorhynchus gorbuscha*) processing by-products were obtained from Dalian Ruichi Food Co., Ltd. (Dalian, China). The fish were imported in headed and eviscerated form; therefore, heads and viscera were not present in the raw material. The by-products consisted primarily of fish bone and skin with adherent residual flesh remaining after fillet removal. The residual meat was mechanically separated from the skeletal and integumentary tissues using a flesh recovery system, followed by washing, dewatering, vacuum packaging, and frozen storage at −20 °C. Bone and skin without adhering flesh were not included in this study, as their valorization requires separate processing strategies beyond the scope of the present work.

Commercially food-grade starches, including potato starch, wheat starch, tapioca starch, maize starch, phosphorylated tapioca starch, hydroxypropyl tapioca starch, tapioca hydroxypropyl distarch phosphate (HDP), and maize acetylated distarch phosphate (MMS), were purchased from Henan Feishi Biotechnology Co., Ltd. (Zhengzhou, China).

### 2.2. Proximate Chemical Composition

The proximate composition analysis of the pink salmon by-product meat was determined according to standard AOAC methods [20]. For protein determination, nitrogen was assayed by the Kjeldahl method, with crude protein derived by applying the conventional 6.25 conversion factor. The lipids were extracted using a Soxhlet apparatus (BUCHI Labortechnik GmbH, Essen, Germany) with diethyl ether as the extraction solvent. Ash content was obtained gravimetrically after a 16 h muffle furnace treatment at 550 °C. In parallel, the phenol-sulfuric acid reaction was employed for the spectrophotometric quantification of total carbohydrates [21].

### 2.3. Amino Acids Analysis

Following acid hydrolysis, amino acid analysis was carried out. Pink salmon by-product meat samples were hydrolysed with 6 mol/L HCl at 110 °C for 24 h in a forced-air drying oven. After the hydrolysis step, the samples were diluted to volume with ultrapure water. Rotary evaporation under vacuum (40–50 °C) was subsequently employed for concentration. The residue was reconstituted in sodium citrate buffer (pH 2.2), and the solution was clarified via a 0.2 µm pore-size membrane. Finally, the clarified filtrates were cryopreserved at −80 °C prior to chromatographic injection. Amino acids were separated by L-8800 automatic amino acid analyser (Hitachi, Tokyo, Japan). Identification and quantification were achieved by comparison with certified reference standards (Sigma-Aldrich, St. Louis, MO, USA) based on retention times and peak area integration. Tryptophan was determined separately following alkaline hydrolysis using the colorimetric method described by Basha and Roberts [22].

### 2.4. Fatty Acid Analysis

The fatty acid composition of pink salmon by-product meat was determined according to a previously reported method [23]. Briefly, 1 g of homogenised sample was subjected to alkaline hydrolysis by adding 0.7 mL of 10 mol/L KOH and 5.3 mL of anhydrous methanol, followed by incubation at 55 °C for 1.5 h. Fatty acid methyl esters (FAMEs) were subsequently prepared by acid-catalysed methylation using 0.58 mL H_2_SO_4_, with further incubation at 55 °C for 1.5 h.

After cooling to room temperature, 3 mL of n-hexane was added to extract the FAMEs. The resulting organic layer was then passed through a suitable membrane filter (organic type) before injection. Chromatographic separation was carried out on a Shimadzu GC-2010 Plus instrument (Shimadzu Corp., Kyoto, Japan), which was fitted with a fused-silica capillary column (60 m × 0.25 mm i.d., 0.25 µm film thickness) and a flame-ionisation detector (FID). Nitrogen was used as the carrier gas. The oven temperature was programmed from 130 °C to 230 °C. Fatty acids were identified by comparison with authenticated FAME standards (Sigma-Aldrich), and their relative contents were calculated from the corresponding peak area percentages.

Cardiovascular health indices were calculated from the fatty acid composition [24]. The atherogenic index (AI) and thrombogenic index (TI) were calculated as follows: AI = (C12:0 + 4 × C14:0 + C16:0)/(ΣPUFA n-6 + ΣPUFA n-3 + ΣMUFA); TI = (C14:0 + C16:0 + C18:0)/(0.5 × ΣMUFA + 0.5 × ΣPUFA n-6 + 3 × ΣPUFA n-3 +ΣPUFA n-3/ΣPUFA n-6).

### 2.5. Preparation of Pink Salmon By-Product Meat Gel

Pink salmon by-product meat gel were prepared according to conventional fish cake manufacturing procedures with slight modifications [25]. Frozen pink salmon processing by-product meat was thawed in a refrigerator at 4 °C for approximately 10 h. A 200 g portion of thawed mince was transferred to the food processor (Kitchen-Aid Trading Co., Ltd., Shanghai, China) and pre-chopped for 30 s.

Sodium chloride (2.0 g, 1%, *w*/*w*), vegetable oil (12.0 g, 6%, *w*/*w*), and seasoning (2.0 g, 1%, *w*/*w*) were added sequentially, and mixed for 5 min at 4 °C. For the treatment groups, 16.0 g (8%, *w*/*w*) of the respective starch types (potato starch, wheat starch, tapioca starch, maize starch, phosphorylated tapioca starch, hydroxypropyl tapioca starch, tapioca hydroxypropyl distarch phosphate or maize acetylated distarch phosphate) was incorporated during chopping. No starch was added to the control group.

Ice water was added intermittently to adjust the final moisture content to approximately 70%. The prepared meat paste was transferred into moulds, and steamed at 100 °C for 20 min. The gels were immediately cooled in ice water and stored at 4 °C prior to analysis.

### 2.6. Texture Profile Analysis

Texture profile analysis (TPA) was performed according to the method of Chang et al. [26]. Gel samples were cut into cubes measuring 20 mm × 20 mm × 20 mm and then tested on a TMS-PRO texture analyser (Food Technology Co., Sterling, VA, USA) using a cylindrical probe (12.7 mm diameter, 35 mm length). The test parameters were set as follows: 30% compression, 60 mm/min crosshead speed, and 5.0 g trigger force. The parameters recorded included hardness, springiness, chewiness, and resilience. Each treatment was analysed using at least six replicates.

### 2.7. Water-Holding Capacity

WHC was determined according to Chang et al. [26]. Approximately 3 g of each gel sample was accurately weighed (W_1_) and wrapped in three layers of filter paper before being placed in a centrifuge tube. Samples were centrifuged at 3000× *g* for 10 min at 4 °C (Hunan Xiangyi Laboratory Instrument Development Co., Changsha, China). Following centrifugation, the samples were reweighed (W_2_). WHC was calculated as follows: WHC (%) = W_2_/W_1_ × 100.

### 2.8. Cooking Loss

Cooking loss was determined according to Li et al. [27], with modifications, to simulate the freeze–reheat cycle experienced by commercial surimi products. Briefly, approximately 20 g of gel samples were frozen at −20 °C for 24 h and their initial mass was recorded as C_1_. The frozen samples were then heated by steaming until the internal temperature attained 70 °C. After cooling to room temperature, the samples were weighed (C_2_). Cooking loss was calculated using the following equation: CL (%) = (C_1_ − C_2_)/C_1_ × 100.

### 2.9. Color Properties

Color properties of all gel samples were measured with a CR-400 colorimeter (Konica Minolta, Osaka, Japan). The color parameters L* (lightness), a* (redness-greenness), and b* (yellowness-blueness) were recorded. The total color difference (ΔE) between each starch-treated sample and the control was calculated as follows:

$$\Delta E=\sqrt{{\left(L_{sample}^{*}-L_{control}^{*}\right)}^{2}+{\left(a_{sample}^{*}-a_{control}^{*}\right)}^{2}+{\left(b_{sample}^{*}-b_{control}^{*}\right)}^{2}}$$

Color differences were subsequently converted to National Bureau of Standards (NBS) units by multiplying ΔE by 0.92 and classified as follows: trace (0–0.5), slight (0.5–1.5), noticeable (1.5–3.0), appreciable (3.0–6.0), much (6.0–12.0), and very much (>12.0) [28].

### 2.10. Scanning Electron Microscopy

The samples were processed following the method described by Chang et al. [26]. The gel specimens were sectioned into thin slices (5 mm × 5 mm × 1 mm) and immediately fixed in 2.5% (*v*/*v*) glutaraldehyde solution at 4 °C for 24 h. Dehydration was achieved through a graded ethanol series (50%, 70%, 90%, and three times with 100%), with each dehydration step lasting 15 min. The slices were freeze-dried (SCIENTZ-12N; Ningbo Scientz Biotechnology Co., Ltd., Ningbo, China), mounted on aluminium stubs, sputter-coated with gold, and observed using a Supra 55 scanning electron microscope (Carl Zeiss, Oberkochen, Germany) operated at an accelerating voltage of 15 kV.

### 2.11. In Vitro Digestibility

The in vitro digestion procedure simulated the oral phase, gastric phase (stomach) and intestinal phase according to Minekus et al. [29]. Simulated salivary fluid, simulated gastric fluid, and simulated intestinal fluid were prepared using the recommended electrolyte stock solutions.

Pink salmon by-product meat or gel samples (15.0 g) were mixed with 10.5 mL simulated salivary fluid, 75 μL 0.3 mol/L CaCl_2_, 1.5 mL α-amylase solution (1500 U/mL), and 2.925 mL distilled water. The pH was adjusted to 7.0 with 1 mol/L HCl or NaOH. The mixture was incubated in a shaking water bath at 37 °C for 2 min. Following the oral phase, 22.5 mL simulated gastric fluid was added to the oral digesta, together with 4.8 mL pepsin solution (3500 U/mL), 15 μL 0.3 mol/L CaCl_2_, and 2.085 mL distilled water. The mixture was homogenized, the pH was rapidly adjusted to 3.0 with 1 mol/L HCl, and the flasks were sealed with Parafilm. Incubation was performed on an orbital shaker at 37 °C and 100 rpm for 2 h. After gastric digestion, the samples were cooled to 25 °C. Then, 33.0 mL simulated intestinal fluid was added to the gastric digesta (60.0 mL), together with 15.0 mL pancreatin solution containing 100 U/mL trypsin and 10 mmol/L bile salt, 120 μL 0.3 mol/L CaCl_2_, and 11.43 mL distilled water. The pH was adjusted to 7.0 with 1 mol/L HCl or NaOH. The mixture was incubated on an orbital shaker at 37 °C and 100 rpm for 2 h. At the end of each phase, reaction mixtures were immediately transferred to an ice-water bath to terminate enzymatic activity.

Following enzymatic hydrolysis, the gastric and intestinal digesta were transferred to centrifuge tubes and centrifuged at 10,000× *g* for 20 min at 4 °C. The resulting pellets were rinsed twice with deionized water and then oven-dried at 105 °C until a constant mass was achieved. The percentage of dry matter digestibility was subsequently derived from the equation given in [30], digestibility (%) = [(M_1_ − M_2_)/M_1_] × 100, where M_1_ is the dry matter weight of the initial sample mass (g) and M_2_ is the weight of the undigested fraction (g).

### 2.12. Statistical Analysis

The experiment was repeated at least three times. Final values are presented as means values ± standard deviation (SD). SPSS software (version 25.0, IBM Corporation, Armonk, NY, USA) was used for statistical analysis of the results. One-way analysis of variance (ANOVA) was performed to evaluate the significance of differences among treatment groups, followed by Tukey’s post hoc test for multiple comparisons at a significance level of *p* < 0.05. The data were reproduced via Origin 2022 software (OriginLab Corporation, Northampton, MA, USA).

## 3. Results and Discussion

### 3.1. Proximate Chemical Composition of Pink Salmon By-Product Meat

The proximate chemical composition of pink salmon processing by-product meat is presented in Table 1. The moisture content was 83.77 ± 1.26%, which was significantly higher than that reported for pink salmon, Atlantic salmon and sockeye salmon fillets [2,31]. This elevated moisture content is primarily attributed to the mechanical separation process [32]. Regarding protein content, the sample exhibited 9.99 ± 0.46% crude protein on a wet weight basis, a value substantially lower than salmon fillets. However, on a dry-weight basis, the protein content reached 61.58%, indicating that the by-product retains a concentrated protein fraction and therefore has considerable potential as a functional food ingredient.

The crude lipid content was 3.73 ± 0.06%, approximately four times higher than that of pink salmon fillets but substantially lower than that of Atlantic and sockeye salmon fillets as shown in Table 1. The ash content was 0.95 ± 0.04%, which was lower than that typically reported for pink salmon and other farmed salmonids. This reduced ash content suggests efficient removal of bone fragments and inorganic material during mechanical separation, thereby improving the texture and palatability of the recovered meat. As expected for marine fish, the carbohydrate content was negligible, which is consistent with previous reports [33].

### 3.2. Amino Acid Profile of Pink Salmon By-Product Meat

The amino acid profile of pink salmon by-product meat is shown in Table 2. A total of 17 amino acids were identified, including 8 essential amino acids (EAAs). The total amino acid (TAA) content was 61.70 ± 0.19 g/100 g on a dry-weight basis. Glutamic acid was the predominant amino acid, exceeding the concentration reported for Atlantic salmon by Fomena Temgoua et al. [34]. Aspartic acid and lysine were the next most abundant amino acids and were also present at higher concentrations than those reported for Atlantic salmon [34]. The predominance of these acidic amino acids contributes to the characteristic umami flavour and enhances protein solubility during food processing.

Among the EAAs, leucine was present at the highest concentration (5.01 ± 0.01 g/100 g), comparable to that reported for Atlantic salmon (4.89 g/100 g) [34]. This was followed by valine and isoleucine. Branched-chain amino acids accounted for 11.27 g/100 g, representing 18.3% of the total amino acid content. These amino acids play a critical role in regulating muscle protein synthesis and influencing glucose homeostasis in humans [35]. The lysine content (5.81 g/100 g) exceeded the FAO/WHO reference pattern [36]. As cereal proteins are deficient in lysine [37], pink salmon protein could effectively complement plant-derived protein sources. Furthermore, the EAA/TAA ratio reached 45.82 ± 0.08%, substantially exceeding the FAO/WHO recommended value of 35%, demonstrating the excellent nutritional quality of the protein.

### 3.3. Fatty Acid Profile of Pink Salmon By-Product Meat

The fatty acid composition of pink salmon by-product meat is presented in Table 3. A total of twenty fatty acids were identified, comprising seven saturated fatty acids (SFAs), six monounsaturated fatty acids (MUFAs), and seven PUFAs. The total SFA content was 5.56 ± 0.02 g/100 g, with palmitic acid (C16:0) being the predominant saturated fatty acid, followed by myristic acid (C14:0) and stearic acid (C18:0). This relatively moderate SFA content suggests a lower cardiovascular risk than that associated with many terrestrial animal fats [24]. The total MUFA content was 5.05 ± 0.78 g/100 g, consisting mainly of oleic acid (C18:1n9c, 1.84 ± 0.05 g/100 g), erucic acid (C20:1, 1.58 ± 0.67 g/100 g), and palmitoleic acid (C16:1, 1.03 ± 0.04 g/100 g). This fatty acid profile may contribute positively to membrane fluidity and metabolic health. The PUFA content reached 10.72 ± 0.13 g/100 g, exceeding that reported for Atlantic salmon (8.95 g/100 g) and many freshwater fish species [38,39]. Among the PUFAs, eicosapentaenoic acid (EPA) and docosahexaenoic acid (DHA) were the predominant components, supporting previous reports that pink salmon is an excellent source of health-promoting unsaturated fatty acids [40].

The n-3/n-6 ratio was 1.34 ± 0.06, which falls within the recommended range (1.0–4.0) for cardiovascular health [41]. Such a ratio has been associated with anti-inflammatory effects and a reduced risk of chronic disease [3]. The calculated AI and TI were 0.44 ± 0.01 and 0.18 ± 0.01, respectively, both substantially lower than those reported for beef, pork, and milk [38]. These findings indicate excellent cardiovascular safety. The fatty acid composition of pink salmon by-product meat supported health claims for functional food development [42].

### 3.4. Textural Property Analysis of Pink Salmon By-Product Meat Gels

**Figure 1:**
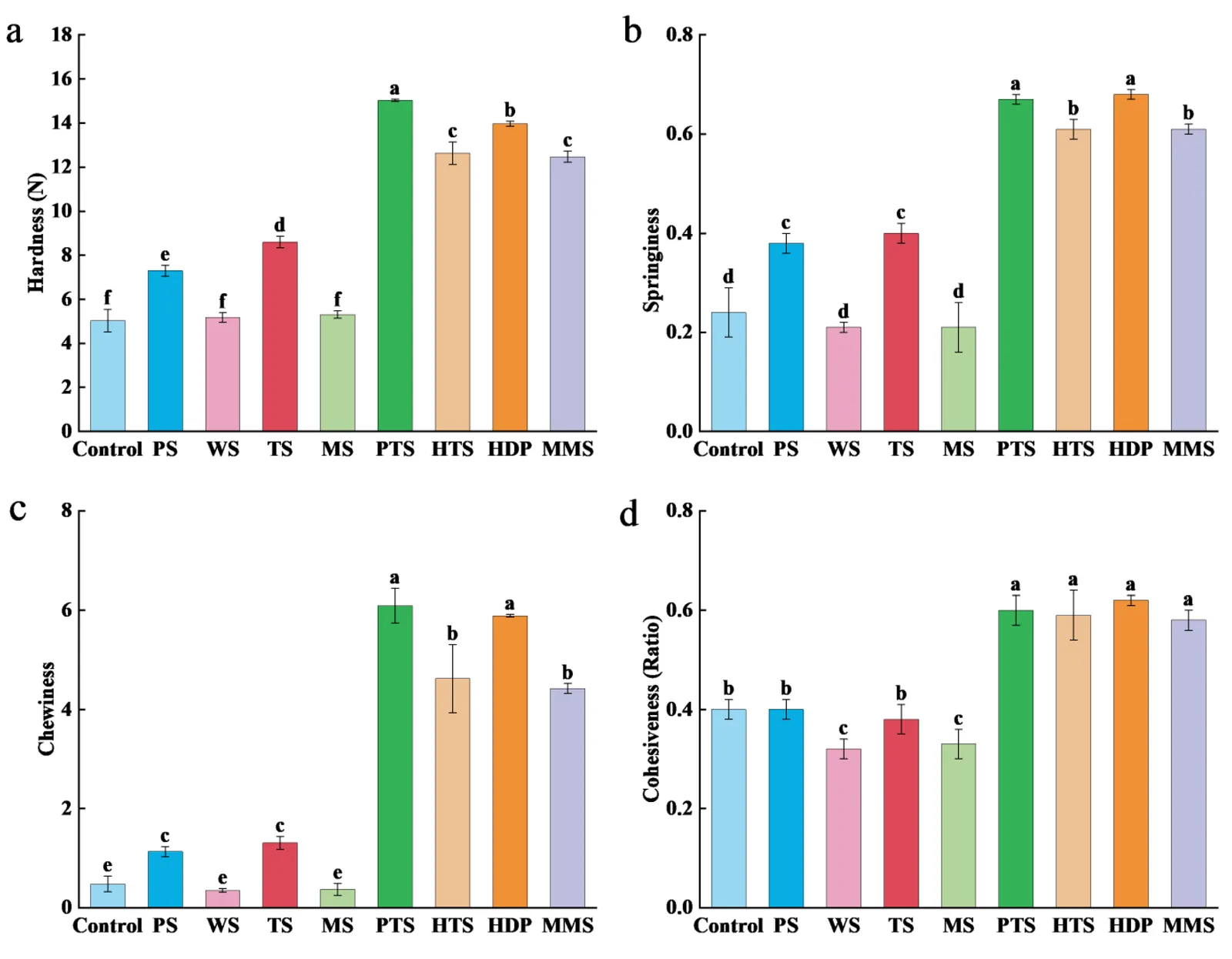
Influence of different starches on the (**a**) hardness; (**b**) springiness; (**c**) chewiness; (**d**) cohesiveness of gel from pink salmon processing by-product meat. Notes: Bars indicate standard deviation from six determinations; different letters denote statistically significant differences (*p* < 0.05). Control, without starch added; PS, potato starch; WS, wheat starch; TS, tapioca starch; MS, maize starch; PTS, phosphorylated tapioca starch; HTS, hydroxypropyl tapioca starch; HDP, tapioca hydroxypropyl distarch phosphate; MMS, maize acetylated distarch phosphate.

TPA is widely employed to evaluate food texture because it characterizes the mechanical response of food materials during large-scale deformation [43]. The TPA results for pink salmon by-product meat gels prepared with different starches are shown in Figure 1.

The control gel, prepared without starch, exhibited a hardness of 5.03 ± 0.51 N and a springiness of 0.24 ± 0.05, indicating a relatively soft gel. This is likely attributable to the presence of water-soluble proteins and lipids in the pink salmon processing by-product meat, which interfere with the protein gelation process and hinder the formation of a stable three-dimensional network. The incorporation of starch significantly influenced the textural properties of the starch-modified gels prepared from pink salmon by-product meat, although the magnitude of improvement depended on both the starch source and modification method. Phosphorylated tapioca starch produced the highest values for most textural parameters (*p* < 0.05), followed by hydroxypropyl tapioca starch and hydroxypropyl distarch phosphate. The phosphate and hydroxypropyl substitutes enhanced starch granule swelling and hydration, thereby promoting more effective filling of voids within the protein matrix and the formation of a denser gel structure [18].

Nevertheless, the textural properties of these gels remained inferior to those of conventional surimi products. For example, *Nemipterus virgatus* surimi supplemented with modified starch exhibited hardness, springiness, cohesiveness, and chewiness values of approximately 32 N, 0.93, 0.81, and 25 N, respectively [44], all substantially higher than those obtained in the present study. This difference most likely reflects the inherent compositional limitations of pink salmon by-product meat.

Among the native starches, tapioca starch produced the greatest improvement in the texture of reconstituted gels, probably owing to its high amylopectin content, relatively large granule size, and superior swelling capacity [45]. Potato starch also improved gel texture because its large granules and high swelling degree capacity contributed to filling the protein network [46]. In contrast, wheat and maize starch produced only modest improvements. Both starches yielded springiness values of 0.21, while chewiness values (0.35 ± 0.04 mJ and 0.37 ± 0.12 mJ, respectively) were significantly lower than those of the control (*p* < 0.05). Their poorer performance is likely due to their relatively high amylose content, which forms rigid and brittle gel structures upon gelatinization, together with their limited swelling capacity [47].

### 3.5. Water-Holding Capacity and Cooking Loss

**Figure 2:**
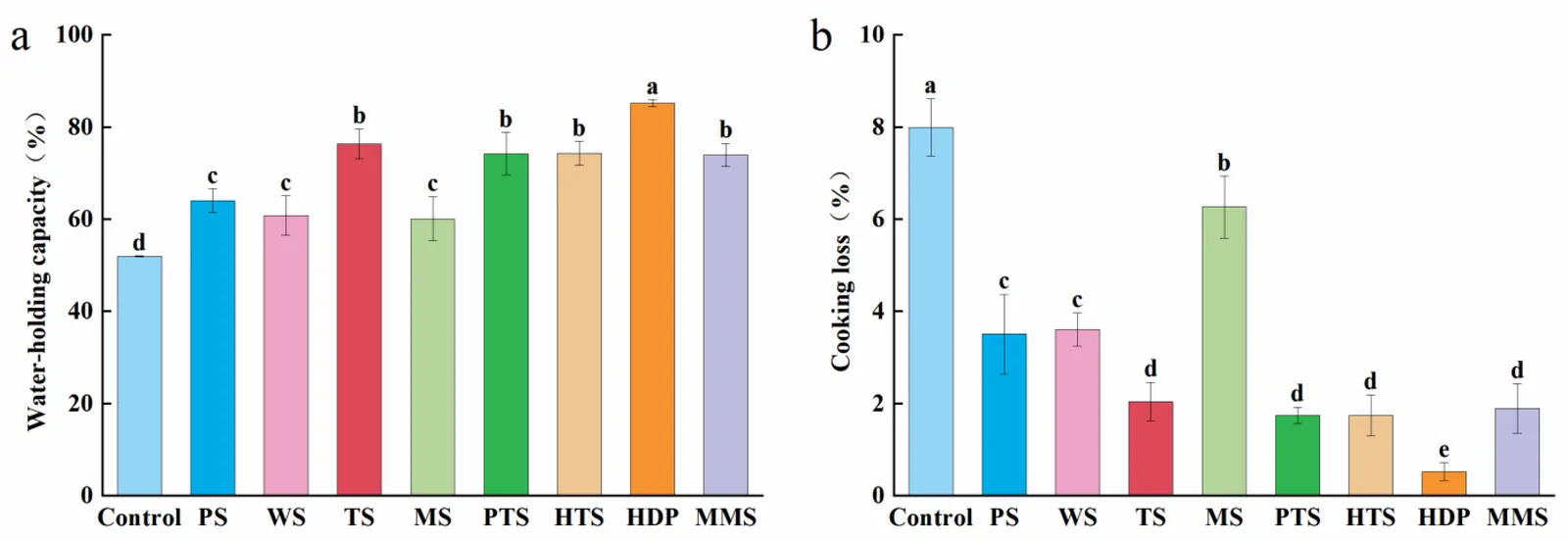
Influence of different starches on the (**a**) water-holding capacity and (**b**) cooking loss of gel from pink salmon processing by-product meat. Notes: Bars indicate standard deviation from triplicate determinations; different letters denote statistically significant differences (*p* < 0.05). Control, without starch added; PS, potato starch; WS, wheat starch; TS, tapioca starch; MS, maize starch; PTS, phosphorylated tapioca starch; HTS, hydroxypropyl tapioca starch; HDP, tapioca hydroxypropyl distarch phosphate; MMS, maize acetylated distarch phosphate.

WHC reflects the ability of the protein network to retain water and is closely related to the structural integrity of surimi gels [48]. The WHC values of the pink salmon by-product meat gels prepared with different starches are presented in Figure 2a.

The modified starches HDP and MMS exhibited higher WHC values than their native counterparts, probably because the hydrophilic substitutes introduced during starch modification enhanced moisture retention within the gel matrix [18]. Previous studies have shown that amylopectin swells more rapidly than amylose at temperatures above 65 °C [49]. Consequently, tapioca starch, which contains a relatively high proportion of amylopectin, displayed superior water retention among the native starches.

As shown in Figure 2b, all starch-containing treatments exhibited significantly lower cooking losses than the control group (*p* < 0.05). These findings indicate that pink salmon by-product meat gels prepared without starch lose substantial amounts of moisture during heating, whereas starch addition effectively immobilises water through granule swelling during protein gelation. Among the starches examined, maize starch resulted in the highest cooking loss, whereas HDP produced the lowest. These differences are most likely attributable to variations in starch swelling behavior and granule rupture during heating [19].

### 3.6. Color of Pink Salmon By-Product Meat Gels

The color characteristics of the pink salmon by-product meat gels are summarized in Table 4. The color parameters were significantly influenced by the varieties of starches. Compared with the control, the L* values decreased for the starch treatments except tapioca starch. Our results were inconsistent with the report of Zhang et al., who reported that starch could improve surimi lightness and reduce a* and b* values [19]. Compared with those of the control group, the a* and b* values of most starch-modified gels prepared from pink salmon by-product meat were significantly increased (*p* < 0.05).

Visual appearance of the gels is presented in Supplementary Figure S1. The control gel exhibited a relatively light color. After starch incorporation, most gels became darker and more yellowish, although the extent of color change depended on the starch type. These visual observations were consistent with the instrumental color measurements (Table 4). Based on the National Bureau of Standards (NBS) classification, gels containing phosphorylated tapioca starch exhibited NBS values over 6.0, categorizing color changes as “much” to the eyes of consumers, according to Pinton et al. [28]. The NBS values ranged between 3.0 and 5.0 for the other modified starch treatments, classifying the changes as “appreciable”. These color changes are likely associated with differences in starch gelatinization and swelling behavior [45]. Moreover, the incorporation of starches significantly improved the water-holding capacity and reduced cooking loss of pink salmon by-product meat gel (Figure 2), which subsequently led to more water retained in the sample. The higher moisture content may have modified light refraction at the gel surface during color measurement, thereby influencing the measured L*, a*, and b* values [50].

### 3.7. Microstructure of Pink Salmon By-Product Meat Gels

**Figure 3:**
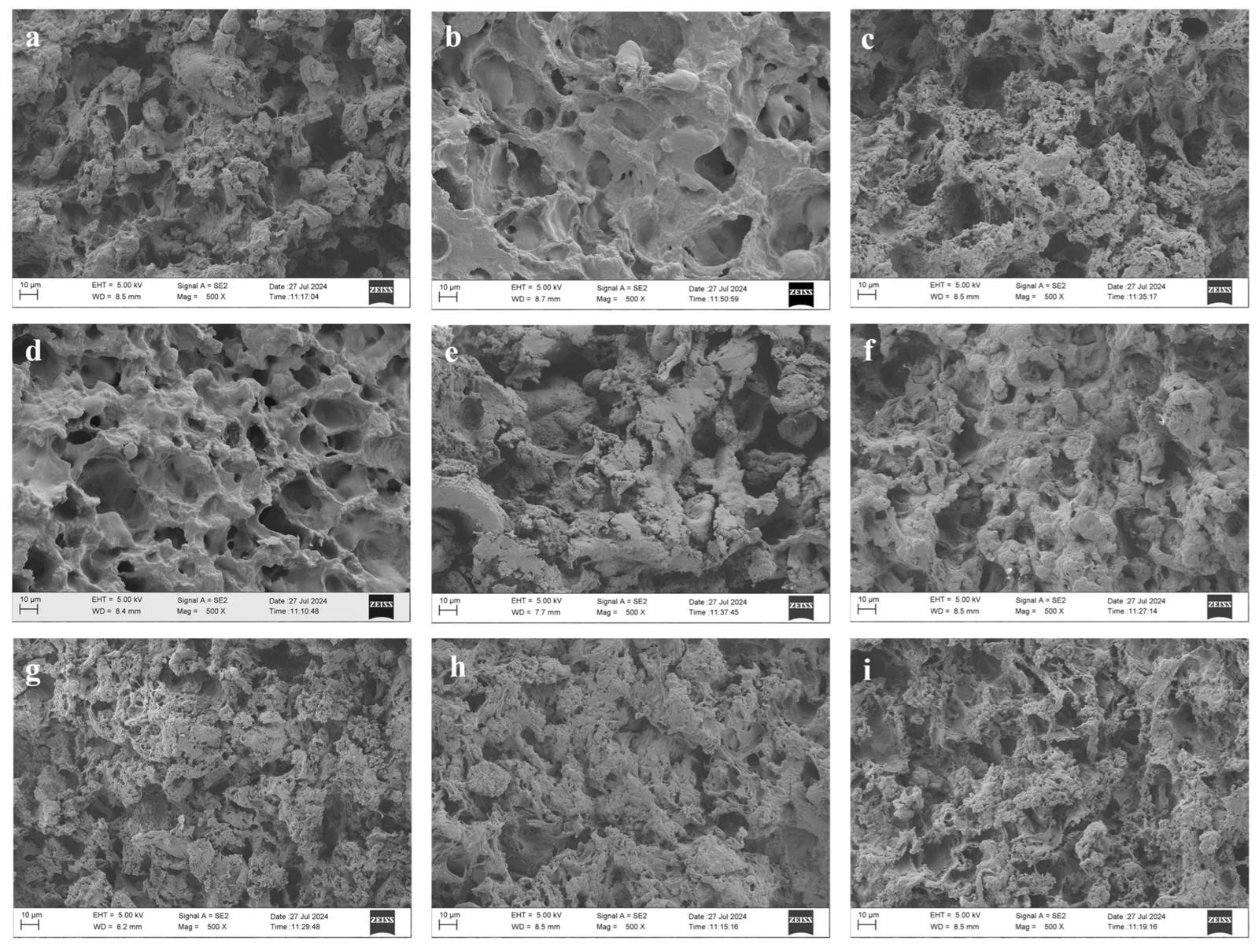
Changes in the microstructure of gels from pink salmon processing by-product meat without or with different starches. Notes: (**a**) Control, without starch added; (**b**) PS, potato starch; (**c**) WS, wheat starch; (**d**) TS, tapioca starch; (**e**) MS, maize starch; (**f**) PTS, phosphorylated tapioca starch; (**g**) HTS, hydroxypropyl tapioca starch; (**h**) HDP, tapioca hydroxypropyl distarch phosphate; (**i**) MMS, maize acetylated distarch phosphate.

The microstructures of the pink salmon by-product meat gels prepared with different starches are shown in Figure 3. The control group exhibited a fragmented matrix characterized by large cavities and a discontinuous surface morphology. This poorly developed structure suggests insufficient unfolding of myofibrillar proteins and limited cross-linking during thermal processing.

The incorporation of native starches markedly altered the microstructure of pink salmon by-product meat gel. Compared with the control, gels containing tapioca starch exhibited a smoother and more cohesive surface; however, the protein network remained less compact than those with the modified starches [17]. Potato starch also produced a denser gel network than the control group, although larger voids were still evident compared with the tapioca starch treatment. In contrast, wheat and maize starch produced only marginal structural enhancement, with their gels retaining relatively loose and brittle network structures.

Among all treatments, the modified starches produced the most pronounced improvements in gel microstructure. In particular, tapioca HDP generated a highly uniform and compact network with substantially reduced pore size. The overall degree of network compactness closely corresponded with the observed gel strength and hardness, indicating that the formation of a denser protein matrix contributed directly to improved enhanced mechanical properties.

The microstructural observations were also consistent with the WHC and cooking loss measurements. Samples with compact and homogeneous gel networks, particularly those containing modified starches, displayed superior water retention and lower cooking losses, as the densely packed gel matrix more effectively entrapped water molecules and restricted their release during heating [51]. Conversely, the loose and porous structures observed in the control, wheat starch, and maize starch treatments were associated with greater cooking losses and reduced water retention, reflecting their limited ability to immobilize moisture within the gel matrix. These concordant trends across microstructure, texture, and water-related properties underscore the interdependence of network architecture and overall gel quality in starch-supplemented pink salmon by-product meat systems.

### 3.8. In Vitro Digestibility of Pink Salmon By-Product Meat and Pink Salmon By-Product Meat Gels

**Figure 4:**
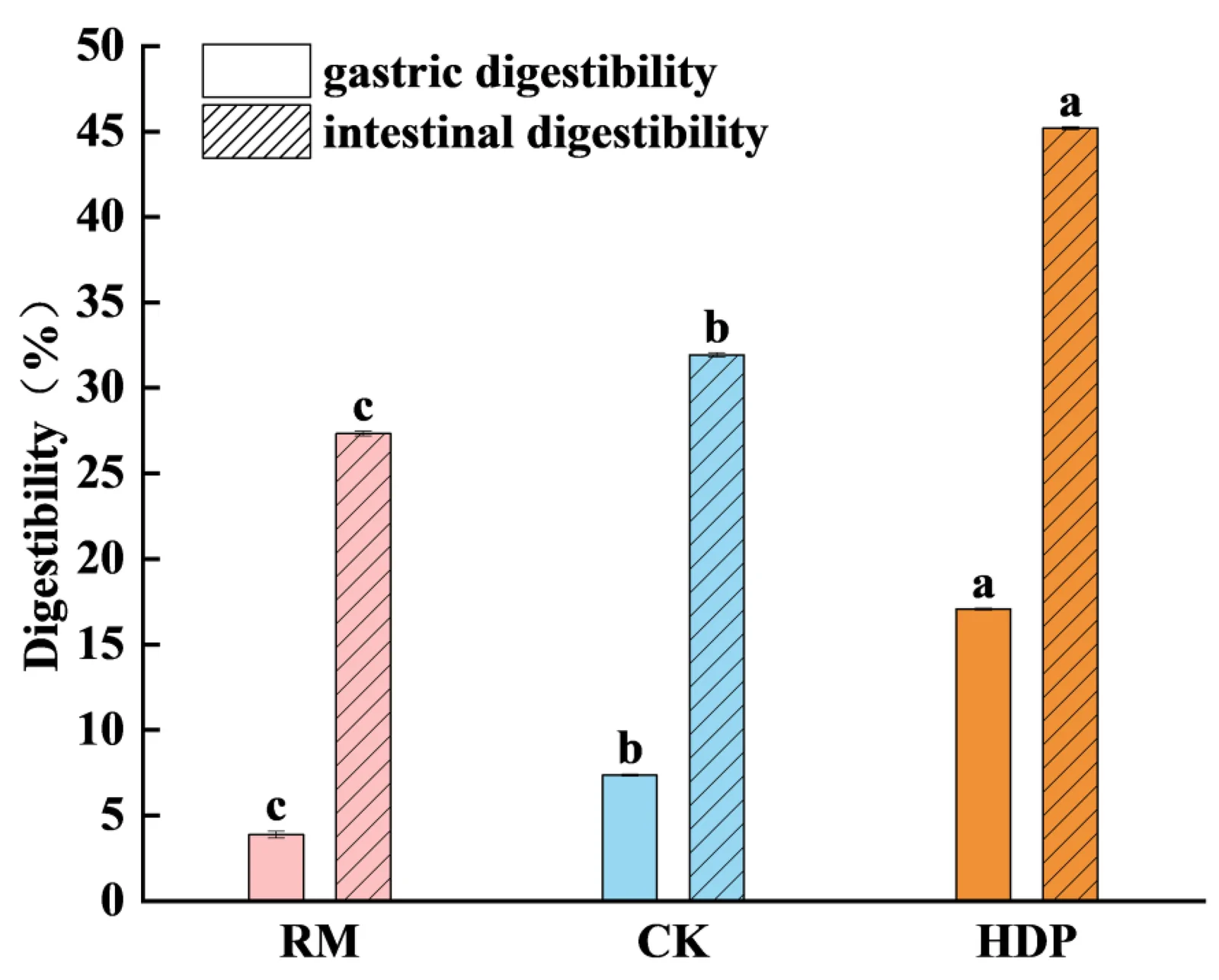
In vitro digestibility of pink salmon by-product meat and gel. Notes: Bars indicate standard deviation from triplicate determinations; different letters denote statistically significant differences between different samples under the same digestion conditions (*p* < 0.05). RM, raw pink salmon by-product meat; CK, pink salmon by-product meat gel without starch added; HDP, pink salmon by-product meat gel with tapioca hydroxypropyl distarch phosphate.

According to the results presented in Section 3.4, Section 3.5, Section 3.6 and Section 3.7, the improvement in the gel properties of pink salmon by-product meat gels following HDP treatment was more pronounced than that observed in the other starch group. This study therefore focused on evaluating the digestive properties of starch-modified gels treated with HDP. The dry matter digestibility of raw pink salmon by-product meat, gel without starch and gel with HDP during the gastric and intestinal digestion phases is presented in Figure 4. Raw material showed the lowest dry matter digestibility (3.89%) in the gastric phase, compared with the intestinal phase, and was significantly less digestible in both phases than the other evaluated groups (*p* < 0.05). These findings indicate that gel formation enhances the digestibility and, consequently, the potential bioavailability of pink salmon byproduct proteins, which is consistent with previous observations reported by Tan et al. [52]. The improved digestibility is likely attributable to the chopping and thermal processing steps, which partially disrupt the native protein structure and increase enzyme accessibility during digestion.

Generally, highly cross-linked and structurally dense protein gels are expected to exhibit lower digestibility [53], which contrasts with the findings of the present study. As shown in Figure 3, HDP produced a denser gel network than that of the control; however, the dry matter digestibility was significantly higher (Figure 4). This is likely attributable to the fact that the starch primarily served as a filler rather than forming tight cross-links with proteins similar to those induced by TGase. Previous studies have similarly demonstrated that modified starches enhance the digestibility of gels, suggesting that modified starch may positively affect the activity of gastrointestinal digestive enzymes [54,55].

## 4. Conclusions

From a nutritional perspective, pink salmon processing by-product meat is an ideal food material and has the impressive profile of high-value nutrients such as good-quality protein and a high amount of PUFA. Starch, especially the modified starch of HDP, improved textural properties of gel from pink salmon by-product meat. Tapioca hydroxypropyl distarch phosphate also increased water-holding capacity, reduced cooking loss rate, and facilitated the formation of a uniform and dense microstructure of the pink salmon by-product meat gels. Additionally, HDP positively impacted the dry matter digestibility of pink salmon by-product meat gels. Despite these improvements, the starch-enhanced gels prepared from pink salmon by-product meat remained dark yellow in color and exhibited a softer texture than conventional commercial surimi products. Therefore, further optimisation is required to improve consumer acceptability. Future studies should investigate the incorporation of suitable food-grade additives or processing technologies to further enhance gel strength, texture, and overall product quality.

## Figures and Tables

**Table 1:** Proximate composition of pink salmon processing by-product meat.

Proximate Composition (% Wet Basis)	Pink Salmon Processing By-Product Meat ^a^	Pink Salmon Fillets ^b^	Atlantic Salmon Fillets ^b^	Sockeye Salmon Fillets ^b^
Moisture	83.77 ± 1.26	79	65–70	70–74
Protein	9.99 ± 0.46	19–20	18–20	21–23
Fat	3.73 ± 0.06	0.8–1.0	10–15	7–8
Ash	0.95 ± 0.04	1.2	1.2–1.5	1.2–1.4
Carbohydrate	0.07 ± 0.01	-	-	-

^a^ Values are shown as the mean and standard deviation of triplicates; ^b^, References [2,31]; -, no available data.

**Table 2:** Amino acid composition (g/100 g, dry weight basis) of pink salmon processing by-product meat.

Amino Acids	Content	Amino Acids	Content
Thr	3.03 ± 0.01	Ser	2.64 ± 0.00
Val	3.33 ± 0.01	Glu	9.00 ± 0.09
Ile	2.93 ± 0.00	Gly	3.30 ± 0.15
Leu	5.01 ± 0.01	Ala	3.93 ± 0.04
Phe	2.67 ± 0.02	Tyr	2.31 ± 0.00
Lys	5.81 ± 0.03	Pro	2.15 ± 0.02
Met	2.07 ± 0.02	TAA	61.70 ± 0.19
Trp	0.69 ± 0.00	EAA	28.27 ± 0.04
His	2.73 ± 0.04	NEAA	33.42 ± 0.15
Arg	3.87 ± 0.03	EAA/TAA (%)	45.82 ± 0.08
Asp	6.23 ± 0.00		

Values are shown as the mean and standard deviation of triplicates; Ala, alanine; Arg, arginine; Asp, aspartic acid; Glu, glutamic acid; Gly, glycine; His, histidine; Leu, leucine; Lys, lysine; Met, methionine; Phe, phenylalanine; Pro, proline; Ser, serine; Thr, threonine; Trp, tryptophan; Tyr, tyrosine; Val, valine; Ile, isoleucine; TAA: total amino acids; EAA: total essential amino acids; NEAA: total nonessential amino acids.

**Table 3:** Fatty acid profile (g/100 g, dry weight basis) of pink salmon processing by-product meat.

Fatty Acids	Content	Fatty Acids	Content
C14:0	1.05 ± 0.05	C18:2n6c	0.32 ± 0.01
C15:0	0.10 ± 0.01	C18:3n3	0.24 ± 0.01
C16:0	2.76 ± 0.12	C20:2	0.07 ± 0.00
C17:0	0.23 ± 0.01	C20:3n3	0.02 ± 0.03
C18:0	0.47 ± 0.02	C20:4n6	4.23 ± 0.16
C21:0	0.74 ± 0.01	C20:5 (EPA)	2.65 ± 0.01
C23:0	0.19 ± 0.19	C22:6 (DHA)	3.19 ± 0.07
Σ SFA	5.56 ± 0.02	Σ PUFA	10.72 ± 0.13
C14:1	0.01 ± 0.01	Σ PUFA n-3	6.10 ± 0.04
C16:1	1.03 ± 0.04	Σ PUFA n-6	4.54 ± 0.17
C17:1	0.12 ± 0.00	Σ PUFA n-3:Σ PUFA n-6	1.34 ± 0.06
C18:1n9c	1.84 ± 0.05	Σ PUFA n-6:Σ PUFA n-3	0.75 ± 0.03
C20:1	1.58 ± 0.67	AI	0.44 ± 0.01
C24:1	0.47 ± 0.01	TI	0.18 ± 0.01
Σ MUFA	5.05 ± 0.78	EPA + DHA	5.84 ± 0.08

Values are shown as the mean and standard deviation of triplicates; SFA, saturated fatty acid; MUFA, monounsaturated fatty acids; PUFA, polyunsaturated fatty acid; AI, atherogenic index; TI, thrombogenic index; EPA, eicosapentaenoic acid; DHA, docosahexaenoic acid.

**Table 4:** Effect of different starches on the color of gel from pink salmon processing by-product meat.

	L*	a*	b*	NBS
Control	73.98 ± 0.22 ^b^	10.57 ± 0.01 ^g^	14.79 ± 0.29 ^f^	-
PS	69.98 ± 0.09 ^f^	11.66 ± 0.03 ^b^	15.52 ± 0.03 ^e^	3.88 ± 0.07 ^c^
WS	73.00 ± 0.09 ^c^	11.30 ± 0.02 ^c^	16.41 ± 0.03 ^c^	1.87 ± 0.02 ^g^
TS	74.32 ± 0.01 ^a^	10.83 ± 0.01 ^e^	16.80 ± 0.01 ^b^	1.89 ± 0.01 ^g^
MS	70.55 ± 0.01 ^e^	10.65 ± 0.03 ^f^	15.62 ± 0.01 ^e^	3.25 ± 0.01 ^f^
PTS	66.93 ± 0.03 ^h^	11.03 ± 0.02 ^d^	16.51 ± 0.03 ^c^	6.69 ± 0.02 ^a^
HTS	70.84 ± 0.03 ^d^	11.99 ± 0.02 ^a^	16.97 ± 0.01 ^ab^	3.75 ± 0.01 ^d^
HDP	69.46 ± 0.02 ^g^	10.85 ± 0.04 ^e^	17.10 ± 0.07 ^a^	4.68 ± 0.04 ^b^
MMS	70.52 ± 0.09 ^e^	10.14 ± 0.02 ^h^	16.11 ± 0.02 ^d^	3.43 ± 0.08 ^e^

Values are shown as the mean and standard deviation of triplicates; lowercase letters indicate statistically significant differences between groups (*p* < 0.05). Control, without starch added; PS, potato starch; WS, wheat starch; TS, tapioca starch; MS, maize starch; PTS, phosphorylated tapioca starch; HTS, hydroxypropyl tapioca starch; HDP, tapioca hydroxypropyl distarch phosphate; MMS, maize acetylated distarch phosphate.

## Data Availability

The original contributions presented in the study are included in the article and Supplementary Materials, further inquiries can be directed to the corresponding author.

## Supplementary Materials

The following supporting information can be downloaded at https://www.mdpi.com/article/10.3390/foods15183259/s1, Figure S1: Appearance of pink salmon by-product meat gels prepared with different starches. Control, without starch; PS, potato starch; WS, wheat starch; TS, tapioca starch; MS, maize starch; PTS, phosphorylated tapioca starch; HTS, hydroxypropyl tapioca starch; HDP, tapioca hydroxypropyl distarch phosphate; MMS, maize acetylated distarch phosphate.
