# Supplementary material for: Valorization of Pink Salmon (Oncorhynchus gorbuscha) Processing By-Products: Nutritional Composition, Starch-Modified Gel Quality and Digestibility
Source: Foods. 2026 Sep 15;15(18):3259. doi: 10.3390/foods15183259 (PMC13605703; doi:10.3390/foods15183259)
Supplement: Supplementary file 1 [file foods-15-03259-s001.zip › foods-4512016-supplementary.pdf]

Supplementary Materials

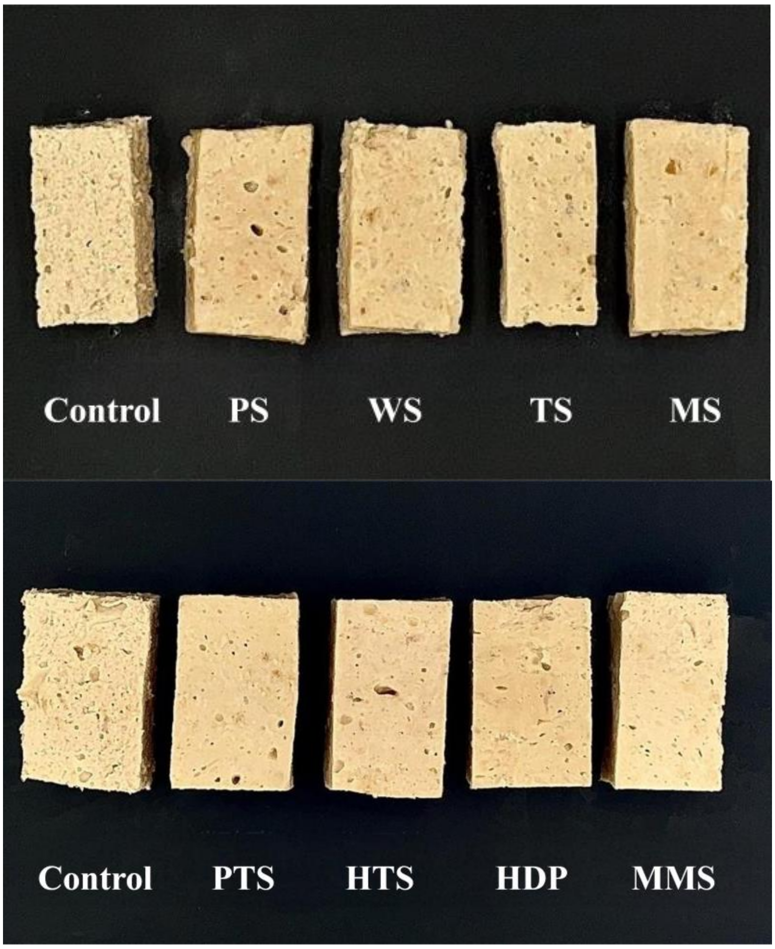

Figure S1. Appearance of pink salmon by-product meat gels prepared with different starches. Control, without starch; PS, potato starch; WS, wheat starch; TS, tapioca starch; MS, maize starch; PTS, phosphorylated tapioca starch; HTS, hydroxypropyl tapioca starch; HDP, tapioca hydroxypropyl distarch phosphate; MMS, maize acetylated distarch phosphate.
